# Embolization of Duodenal Hematoma Post-Endoscopy in Noonan Syndrome: A Novel Management Approach

**DOI:** 10.1097/PG9.0000000000000050

**Published:** 2021-03-08

**Authors:** Samuel C. Ellison, Muddassir Rashid, Looi C. Ee, Robert N. Lopez

**Affiliations:** From the * Department of Gastroenterology, Hepatology and Liver Transplantation, Queensland Children’s Hospital, South Brisbane, QLD, Australia; and; † Department of Radiology, Queensland Children’s Hospital, South Brisbane, QLD, Australia.

## CASE REPORT

A 6-year-old girl with Noonan Syndrome (confirmed SOS1 mutation), underwent elective upper gastrointestinal endoscopy for symptoms of gastro-oesophageal reflux disease. She had a normal aorta and no known bleeding diathesis.

Endoscopy was undertaken with a 8.9 mm outer diameter pediatric gastroscope. Direct visualization was unremarkable, and 2 biopsies each were obtained from the proximal and distal esophagus and 3 from the second part of the duodenum (D2) with standard biopsy forceps (2.2 mm). The procedure was uneventful, and the patient discharged home that same morning.

She presented to the Emergency Department 48 hours later with abdominal pain, vomiting, fever, and tachycardia. Abdominal examination revealed generalized tenderness without peritonism. Hemoglobin dropped from 116 g/L to 97 g/L, with a normal platelet count (216 × 10^9^/L) and normal coagulation profile (INR 1.2, prothrombin time 13 seconds, APTT 28 seconds, fibrinogen 2.7 g/L). D-dimer was elevated at 1.06. Electrolyte profile, liver biochemistry, and serum lipase were unremarkable, while lactate on venous blood gas was 0.5.

**FIGURE 1.**
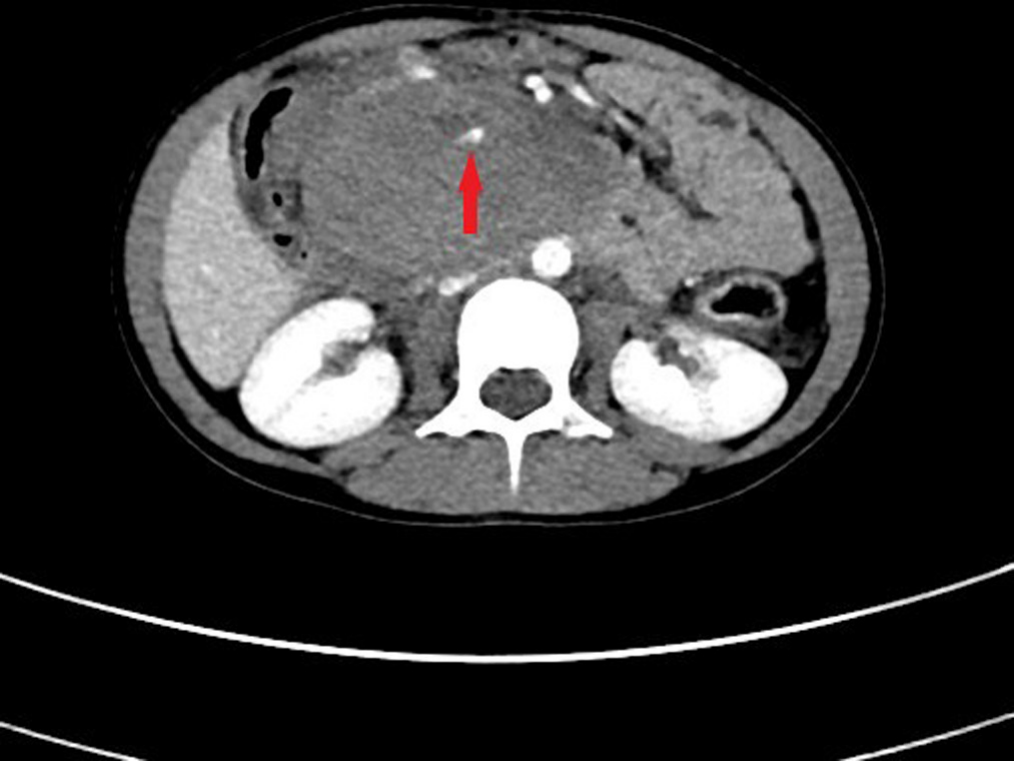
Axial CT image of abdomen demonstrates a large intramural hematoma in second and third part of duodenum with focal contrast extravasation (arrow) consistent with active bleeding.

**FIGURE 2.**
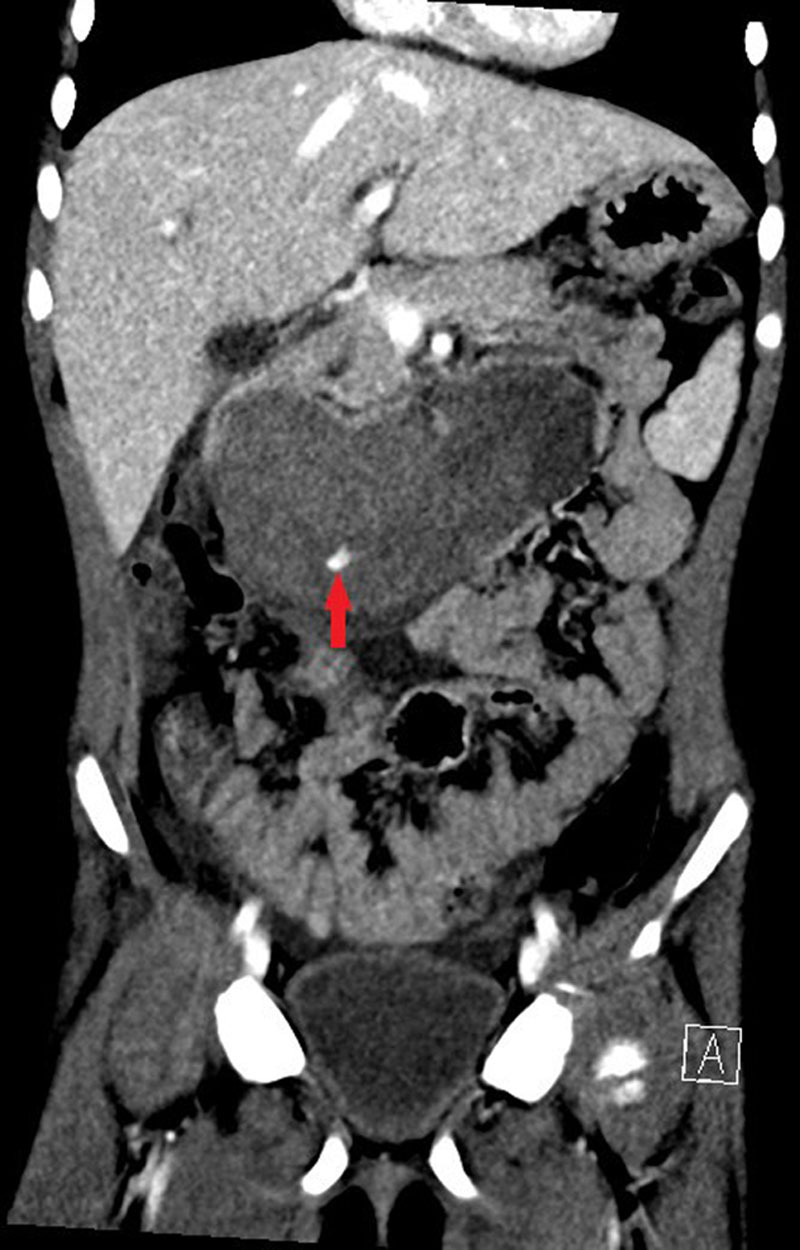
Coronal CT image of abdomen demonstrates a large intramural hematoma in second and third part of duodenum with focal contrast extravasation (arrow) consistent with active bleeding.

Abdominal ultrasound revealed a heterogenous mass conforming to the region of D2 tapering to the duodenojejunal flexure, where normal, collapsed jejunum was demonstrated. The mass measured approximately 9 × 5 × 5 cm and appeared avascular centrally, consistent with duodenal hematoma. Furthermore, there was a moderate volume of free-fluid noted within the pelvis and iliac fossae. A computed tomography (CT) was then undertaken, which confirmed the presence of a hematoma, involving D2-D4 segments, with a maximum dimension of 100 × 40 × 45 mm. There was active contrast extravasation into D2/D3 segment during the portal venous phase of the study, and pooling during the delayed phase, consistent with acute bleeding (Figs. 1 and 2).

**FIGURE 3.**
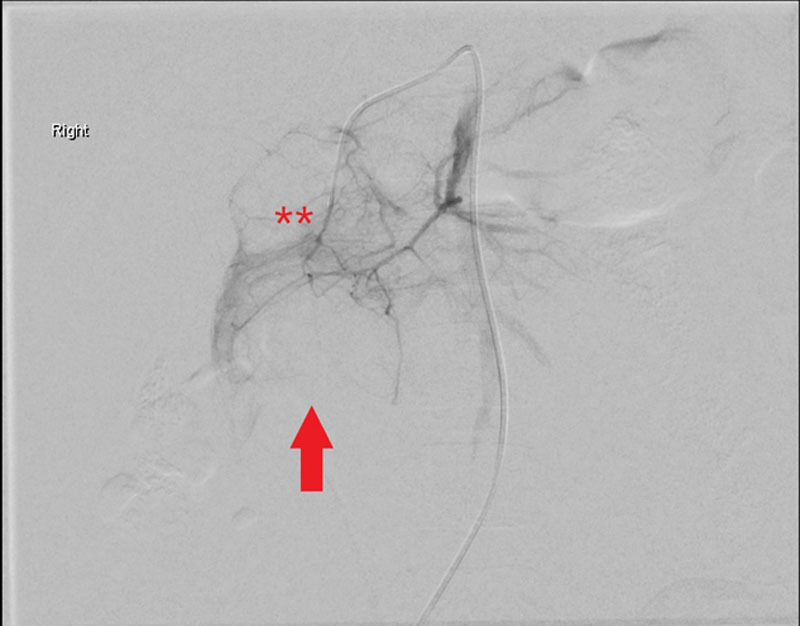
Selective digital subtraction angiogram from coeliac artery with microcatheter in superior pancreaticoduodenal artery outline pancreaticoduodenal arcade. No active bleeding was seen, note the large extrinsic bulge (arrow head) on duodenum representing intramural hematoma with small duodenal branches (**) from arcade running toward hematoma.

**FIGURE 4.**
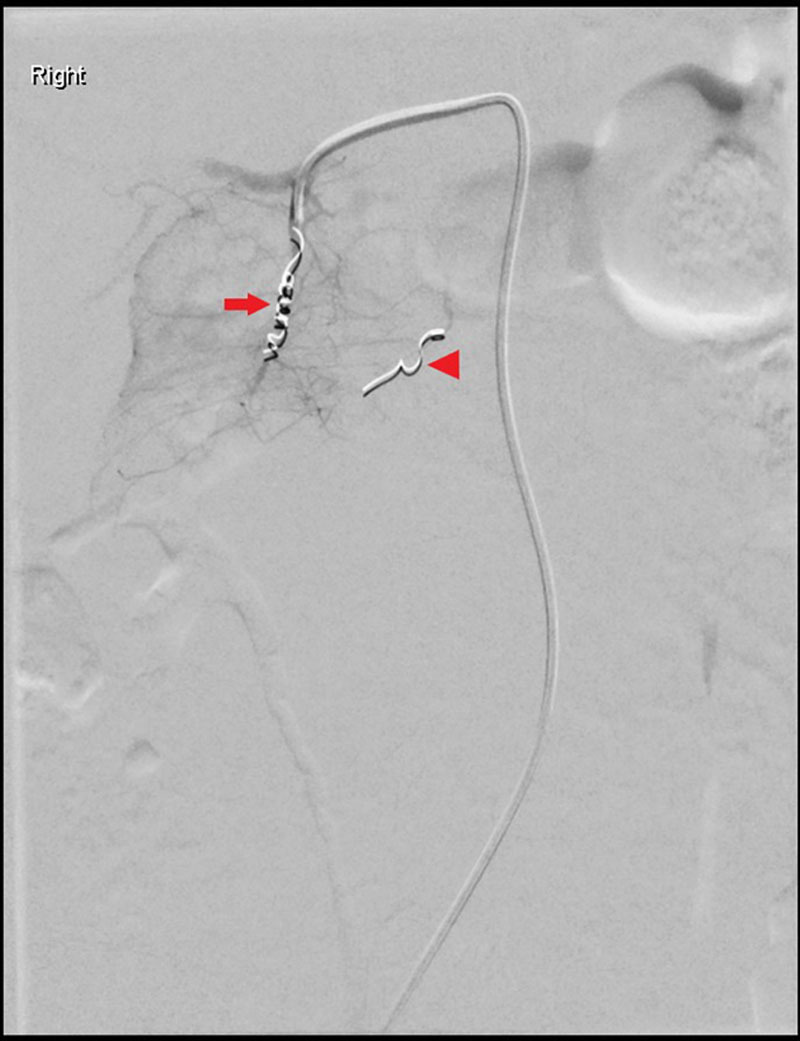
Final selective digital subtraction angiogram demonstrates coils in superior pancreaticoduodenal (arrow) and inferior pancreaticoduodenal (arrow head) branches with the cessation of flow in the duodenal branches, which were seen running toward intramural hematoma on initial angiogram.

Given evidence of active bleeding on CT scan as illustrated by contrast extravasation and intraperitoneal free fluid, in the context of a drop in the patient’s hemoglobin, endovascular embolization of the bleeding vessel was agreed upon, following consultation with the interventional radiology and surgical services. This was performed within 24 hours of the patient representing to hospital. The superior pancreaticoduodenal artery was selectively catheterized with a microcatheter via the celiac trunk. Selective angiography revealed vasospasm in a small muscular branch within the wall of the duodenum running toward a bulge, which denoted a large intramural hematoma, seen in D2 and D3, and supplied by small duodenal branches of the pancreaticoduodenal arcade (Fig. 3). Angiography revealed no evidence of active bleeding. The pancreaticoduodenal arcade supplying D2/3 was embolized with three 2 × 3 mm tornado embolization coils (COOK Medical), involving occlusion of the superior (front door) and inferior (back door) pancreaticoduodenal branches (Fig 4).

Postembolization, the patient was managed on enteral rest, total parenteral nutrition, and proton-pump inhibition. Repeat CT performed 5 days postendoscopy showed interval reduction of the hematoma. A barium swallow 4 weeks postendoscopy showed normal passage of contrast through the duodenum.

Over the course of 4 weeks, the patient was transitioned onto full oral feeds before discharge home. Further investigation, performed following consultation with the hematology service and after recovery, showed normal levels of factor VIII, IX, XI, and XII, normal platelet aggregation and function, and normal von-Willebrand profile.

## DISCUSSION

Duodenal hematoma is an uncommon complication of upper gastrointestinal endoscopy occurring at a rate of 0.05–0.06% (1, 2), as compared to the overall risk of bleeding, which is 0.3% (3). This procedural complication occurs most commonly in children (4, 5). As of 2016, there were 47 published cases of duodenal hematoma, which had occurred in pediatric patients following endoscopic biopsy (6).

Apart from the obtainment of mucosal biopsies, therapeutic interventions and coagulation disorders are known risks for the development of hematoma following upper gastrointestinal endoscopy (5). Children with bone marrow transplants and those suspected of having GVHD are additionally recognized as being high risk (5). It has been suggested that aspects of duodenal anatomy, including its relative immobility (due to its retroperitoneal location) and its rich submucosal blood supply may predispose to bleeding when a shearing force, such as biopsy forceps, has been applied (2, 6, 7). Additionally, it has been suggested that method of sedation and supine positioning may increase the risk of hematoma formation (2). Patients typically present with abdominal pain and vomiting within 72 hours of their endoscopy (2). Proposed management consists of nonoperative care with nutritional support (5).

Noonan syndrome is an autosomal dominant, genetic condition, which has a prevalence of 1 in 1000–2500 births (8). It is manifest by a conglomeration of distinctive facial features, developmental delay, short stature, chest deformity, congenital heart disease, renal anomalies, lymphatic malformations, and bleeding difficulties (8, 9). There are six reports of patients with Noonan syndrome developing duodenal hematomas. These consist of an adult patient postduodenal biopsy (10), children postduodenal biopsy (6, 11–13) as well as a spontaneous occurrence in a child (14).

Noonan syndrome has been associated with hemostatic disorders, including factor XI deficiency, thrombocytopenia, and abnormal platelet dysfunction (15). The prevalence of coagulation and platelet disorders is high in Noonan syndrome (87–93%); however, only half present with bleeding symptoms (11). This suggests that coagulation results do not always correlate with a predisposition to easy bleeding (16). In the present case, the patient had no history of easy bleeding nor significant hemorrhage and her extensive hematological screen was essentially normal.

A recent publication provides a useful approach to high-risk endoscopy in children (17). The authors highlight patients with leukemia or hematopoietic stem cell transplantation as being at increased risk of bleeding, including for duodenal hematoma. No similar caution is issued with regards to patients with Noonan syndrome.

Literature based on anecdotal evidence predominantly report expectant, nonoperative management of duodenal hematomas occurring postbiopsy (5). Surgical management is usually reserved for patients who do not improve with conservative management (4, 7). The decision to proceed with angiography in the present case was based on the large size of hematoma and active bleeding seen on CT in the context of a postprocedural complication. Vasospasm is an indirect sign of vascular injury when direct signs of active bleed are not visible on angiography (18)—an invasive procedure with inherent risk. Embolization of mesenteric arterial branches poses risk of bowel ischemia and should be performed carefully to minimize nontarget embolization. Given the rich collateral blood supply to the duodenum, risk of procedural ischemia was judged to be low in the present case.

While the patient’s time to recovery and discharge are similar to those previously reported, it is conceivable that her admission would have been longer had the hematoma been bigger—an outcome likely had embolization not occurred. This case provides the first-ever description of radiologically guided embolization of an actively bleeding duodenal hematoma occurring after endoscopy.

The increased prevalence of hemostatic abnormalities in children with Noonan syndrome likely warrants extra consideration before and during intestinal endoscopy. Practical changes that could be considered include use of a smaller endoscope (and biopsy forceps) and questioning the necessity for duodenal biopsies at all (6) in children with Noonan syndrome. Intramural duodenal hematoma may be the first presentation of an underlying bleeding disorder, suggesting the need for suspicion in those with Noonan syndrome even without a significant bleeding history (12). Indeed, it has been recommended that patients with Noonan syndrome undergo a hematological review before any invasive procedure (9).

While the nonoperative approach to the care of patients who have experienced a duodenal hematoma following endoscopic biopsy has been the primary management approach to-date, an interventional radiological option could be considered when there is concern about ongoing bleeding and or hemodynamic instability.
